# Editorial: Insect Olfactory Proteins (From Gene Identification to Functional Characterization), Volume II

**DOI:** 10.3389/fphys.2022.858728

**Published:** 2022-03-02

**Authors:** Peng He, Yang Liu, J. Joe Hull, Ya-Nan Zhang, Jin Zhang, Xiao-Jiao Guo, Wei Xu

**Affiliations:** ^1^State Key Laboratory Breeding Base of Green Pesticide and Agricultural Bioengineering, Key Laboratory of Green Pesticide and Agricultural Bioengineering, Ministry of Education, Guizhou University, Guiyang, China; ^2^State Key Laboratory for Biology of Plant Diseases and Insect Pests, Institute of Plant Protection, Chinese Academy of Agricultural Sciences, Beijing, China; ^3^Pest Management and Biocontrol Research Unit, US Arid Land Agricultural Research Center, USDA Agricultural Research Services, Maricopa, AZ, United States; ^4^Anhui Key Laboratory of Pollutant Sensitive Materials and Environmental Remediation, College of Life Sciences, Huaibei Normal University, Huaibei, China; ^5^Department of Evolutionary Neuroethology, Max Planck Institute for Chemical Ecology, Jena, Germany; ^6^State Key Laboratory of Integrated Management of Pest Insects and Rodents, Institute of Zoology, Chinese Academy of Sciences, Beijing, China; ^7^Agricultural Sciences, Murdoch University, Murdoch, WA, Australia

**Keywords:** odorant-binding proteins, chemosensory proteins, odorant receptors, ionotropic receptors, odorant-degrading enzymes

The insect olfactory system is a highly evolved network of proteins that are essential for perception of the local environment, which includes communication with conspecific individuals and determining the location of suitable hosts and mating partners. The olfactory receptor neurons (ORNs) that comprise the detection component of the system are housed in the hair-like sensilla that line the antennae (i.e., the predominate olfactory organ). To date, a number of genes essential for differentiating key information from the complex odorant milieu of the environment have been identified. This filtering system includes: odorant carrier proteins, such as odorant-binding proteins (OBPs) and chemosensory proteins (CSPs), receptor proteins, which consist of odorant receptors (ORs) and ionotropic receptors (IRs), and odorant-degrading enzymes (ODEs) including various enzymes such as carboxylesterases, P450, oxidases, and so on. Although advances in sequencing technologies have driven a surge in the number of olfactory genes identified, our understanding of their functionality and the molecular mechanisms underlying their respective interactions remains limited. This Topic, an expansion of the Insect Olfactory Proteins (From Gene Identification to Functional Characterization) topic, seeks to address this limitation by highlighting research on a diversity of insect olfactory systems. In total, we have collected 18 papers representing species from three insect orders (8 Lepidoptera, 6 Coleoptera, and 4 Hemiptera) that impact the agricultural, forest, and medical fields.

In addition to the studies elucidating components of the first step in olfaction (i.e., detection), we have also included two studies examining the neural-based second step—perception. In the paper by Chen et al. the authors measured the electrophysiological responses of *Spodoptera frugiperda* labial palps to CO_2_ and host volatiles. In the other paper, Liu J. et al. generated 3D digital reconstructions of the antennal lobe macroglomerular complex of males from two sibling moth species (*Ectropis obliqua* and *Ectropis grisescens*) that respond to opposite sex pheromone combinations. Volumetric differences in the anterior-lateral glomerulus and posterior-ventral glomerulus of the two species suggest a possible reason for the differing biological responses to sex pheromone compounds.

## Transcriptome-Based Identification and Expression Pattern of Novel Olfactory-Related Genes

Improvements in RNA sequencing and subsequent data processing/analyses have made transcriptomes more economical, which has not only facilitated identification of olfactory-related genes but also made it feasible to assess transcript expression across a range of tissues, development stages, and conditions. In this Topic, Zhu et al. generated sex specific antennal transcriptomes for a bark beetle (*Scolytus schevyrewi*) fruit tree pest. In addition to identifying 47 ORs, 22 IRs, 22 OBPs, and 11 CSPs, their study also used RT-PCR to examine the tissue expression profile of the identified OBPs and CSPs. In a different study, Yi et al. screened adult antennal transcriptomes from another beetle pest (*Holotrichia parallela*) for a specific class of ODE, carboxylesterases (CXEs). Homologous BLAST analyses allowed the authors to identify 20 candidate CXEs, seven of which were found via RT-qPCR to exhibit antennae-biased expression. Similarly, Liu H. et al. identified 17 candidate CXE glutathione-S-transferases (GSTs) from antennal transcriptomes of the Indian meal moth (*Plodia interpunctella*), one of which *PiGSTd1* was predominantly expressed in male antennae and was shown to efficiently degrade a sex pheromone component as well as some host odorants.

Using an antenna-specific transcriptome from the greater wax moth (*Galleria mellonella*), Jiang et al. identified 102 olfactory genes, including 21 OBPs, 18 CSPs, 43 ORs, 18 IRs, and 2 SNMPs, and examined the tissue expression profile of a subset of the genes. In contrast, Zhang, X. et al. used broader transcriptomic datasets (larval heads and integuments) to identify 15 OBPs, 6 CSPs, 2 ORs, 14 IRs, and 1 SNMP in *Endoclita signifier*, a lepidopteran pest of eucalyptus trees. Elevated expression of a subset (5 OBPs, 2 CSPs, and 1 OR) of the genes in larval heads led the authors of the study to posit potential olfactory roles. In a comparative study of sibling beetle weevil (*Eucryptorrhynchus scrobiculatus* and *Eucryptorrhynchus brandti*) transcriptomes, Wang Q. et al. identified 12 CSPs and found that *CSP7, 8*, and *9* in both species are mainly expressed in adult antennae. Additionally, EscrCSP8a and EbraCSP8 shared relatively low sequence identity and showed distinct binding affinities with (1R)-(+)-alpha-pinene, (–)-beta-caryophyllene, and beta-elemen *via* docking analysis.

OBP expression profiles are frequently differentiated by phenotypic-dependent functional roles. In support of this, Zhang S. et al. used transcriptomic datasets from the grain aphid (*Sitobion miscanthi*) to examine the temporal expression of five OBPs including three previously identified as aphid alarm pheromone (E-β-farnesene, EBF) binding proteins. Intriguingly, they found relatively stable and high expression of *OBP9* in adults of both wing morphs and that expression was upregulated in response to EBF induction suggesting this protein may be a crucial molecule for EBF recognition in aphids. In contrast, the effects of EBF on *OBP7* were limited to only winged adults and that *OBP3* was not induced in either wing type.

Wang L. et al. similarly used transcriptomic and RT-qPCR analyses across various tissues to examine OBP expression in two wing morphs of the soybean aphid (*Aphis glycines*). In this aphid species, all 15 OBPs, including three novel OBPs, exhibited varied expression profiles across tissues. The expression of seven OBPs, however, were significantly higher in winged adults than wingless adults, and *OBP6* were differentially expressed between the two wing phenotypes.

## Typical and Atypical Functional Roles of OBPs and CSPs

Although the olfactory function of OBPs and CSPs is frequently indicated by elevated and/or specific expression in antennae, the role of these proteins in binding biologically relevant odorants has also been demonstrated both *in vivo* and *in vitro*. Using an *in vitro* fluorescence-binding assay, Li et al. measured the binding affinities of a subset of recombinant OBPs and CSPs from the coleopteran pest *Galeruca daurica* (Joannis) for host-derived odorants. Two OBPs (*6* and *15*) and two CSPs (*4* and *5*) were found to bind multiple host plant odorants. Furthermore, RNAi-mediated knockdown of *OBP15* and *CSP5* reduced electroantennogram (EAG) responses in female adults to a panel of host volatiles. Similarly, Wang Z. et al. show that expression of *CSP15* in the brown marmorated stink bug (*Halyomorpha halys*) is likewise antennae dominant and *in vitro* assays revealed high binding affinities for EAG-active host volatiles β-ionone, cis-3-hexen-1-yl benzoate, and methyl (2E,4E,6Z)-decatrienoate.

In addition to olfaction, OBPs and CSPs have also been implicated in gustation, vision, and insecticide responses. Guo et al. comprehensively analyzed the spatiotemporal expression profile of four silkmoth (*Bombyx mori*) OBPs—two pheromone-binding proteins (PBP) and two general odorant-binding proteins (GOBPs). Expression of the respective transcripts varied with age and all were found expressed in non-olfactory tissues including the early embryo, brain, and silk gland. Furthermore, expression in non-olfactory tissues was induced in response to abamectin exposure. Based on these results, the authors posit novel roles for OBPs in pesticide binding and suggest they have additional functions beyond antennal sex pheromone detection.

## Molecular and Functional Characterization of ORs

Functional ORs are a dimeric complex between specific, narrowly tuned ORs and Orco, a highly conserved OR co-receptor. In this Topic, Zhang, Liu, et al. cloned the *Orco* gene from the white-spotted flower chafer (*Protaetia brevitarsis*) and found that silencing the gene impaired EAG responses to an aggregation pheromone and impeded identification of fresh food sources. In contrast, He et al. focused on characterizing a pair of a narrowly tuned ORs from the potato tuber moth (*Phthorimaea operculella*), both of which were specifically activated by two of the major sex pheromone components, suggesting that they function *in vivo* as pheromone receptors (PRs). Ji et al. similarly used an *in vitro Xenopus* expression system to examine OR ligand specificity and found that OR6 from the red palm weevil (*Rhynchophorus ferrugineus*) is narrowly tuned to α-pinene.

## Overview and Characterization of ODEs

Although ODEs consist of multiple enzyme families, esterases are the most well-studied to date. In their review of lepidopteran antennal ODEs, Godoy et al. highlight evolutionary relationships as well as key structural/functional features and discuss efforts to target ODEs for inhibition as a potential integrated pest management strategy. In contrast, Liu H. et al. focus on characterization of a single, highly expressed, male antennae dominant GST from the Indian meal moth (*P. interpunctella*) that efficiently degraded both acetate sex pheromones and a series of aldehyde host volatiles.

## Prospects, Challenges, and Potential Applications to Pest Management

Even though advances in sequencing technologies have facilitated the identification of numerous genes critical to insect olfaction, incorporation and successful implementation of this knowledge into pest management remains challenging. Liu F. et al. provide a broad overview of olfaction in hematophagous hemipterans from the current olfactory mechanism paradigm to effects on host-seeking behavior and finally chemosensation-based management possibilities. They suggest that a combination of PULL-PUSH-MASK could be the best solution based on classic chemical ecology using chemical lures, repellents, and confusants. Further investment in reverse chemical ecology will continue to offer the possibility of accurately predicting critical chemical structures based on crucial olfactory genes. However, pairing that approach with novel gene editing/knockdown methods such as CRISPR and RNAi, could lead to the development of critical breakthroughs that allow olfaction-based methods of pest management to replace current reliance on chemical pesticides.

## Concluding Remarks

We truly appreciate all authors' contributions to this Topic, which illustrate the diversity of studies currently underway on insect olfactory proteins. We also thank all reviewers and editors who assisted us and provided thorough comments and invaluable suggestions, as well as the Frontiers editorial team for its support on the Topic management.

## Author Contributions

PH, YL, JH, Y-NZ, JZ, X-JG, and WX wrote, edited, and finalized this document. All authors contributed to the article and approved the submitted version.

## Funding

This report was supported by the National Natural Science Foundation of China (Grant No. 31860617 for PH), the Natural Science Foundation of Guizhou Province of China [Grant No. QKH-J (2020)1Y077 for PH], the Talent Cultivation Program at Guizhou University [(2019)05 for PH], the Program of Introducing Talents of Discipline to Universities of China (111 Program, D20023), and the Frontiers Science Center for Asymmetric Synthesis and Medicinal Molecules, Department of Education, Guizhou Province [Qianjiaohe KY number (2020) 004].

## Conflict of Interest

The authors declare that the research was conducted in the absence of any commercial or financial relationships that could be construed as a potential conflict of interest.

## Publisher's Note

All claims expressed in this article are solely those of the authors and do not necessarily represent those of their affiliated organizations, or those of the publisher, the editors and the reviewers. Any product that may be evaluated in this article, or claim that may be made by its manufacturer, is not guaranteed or endorsed by the publisher.

